# Sociodemographic factors and environmental workers’ knowledge of the impact of awareness creation on sustainable disposal of solid wastes

**DOI:** 10.1016/j.heliyon.2023.e18122

**Published:** 2023-07-17

**Authors:** Uba James Uba, Etim Nkanu Efut, Udumo Bassey Obeten, Edung Etim Asuquo, James Connie Uba

**Affiliations:** a School of General Studies, Federal Polytechnic Ugep, Cross River State, Nigeria; b Department of Environmental Education, Faculty of Arts and Social Science Education, University of Calabar, Nigeria; c Department of Biochemistry, Nutrition and Food Science, University of Calabar, Nigeria

**Keywords:** Sociodemographic factors, Environmental workers knowledge, Awareness creation, Sustainable disposal, Solid waste materials

## Abstract

This study examined sociodemographic factors and environmental workers' knowledge of the impact of awareness-creation on the sustainable disposal of solid waste in Cross River State, Nigeria. To achieve the purpose of the study, two research questions and two hypotheses guided the study. The sequential explanatory mixed-method design was adopted in the study. The population for the study was 331 respondents, consisting of 120 staff and 211 government-approved service providers from the Cross-River State Ministry of Environment. The population also comprised the sample size of the study. The total enumeration sampling technique was employed. A 42-item structured questionnaire titled “Questionnaire Instrument on Sociodemographic Factors and Environmental Workers’ Knowledge of the Impact of Awareness Creation on Sustainable Disposal of Solid Wastes” (QISFEWKIACSDSW) and a Focus Group Discussion Schedule (FGDS) were the instruments used for data collection. The instruments were face validated by three experts: two from Social Science Education and one from the Measurement and Evaluation Unit of the Department of Science Education, University of Nigeria, Nsukka. The Cronbach Alpha Reliability Method was used to determine the internal consistency of the instrument. Data collected from the questionnaire was analyzed using mean and standard deviation for the research questions while independent sampled *t*-test statistic was used to test the null hypotheses at a 0.05 level of significance and the FGDS was used to supplement the findings of the quantitative analysis. Findings of the study revealed that, to a high extent, awareness creation based on location and gender can contribute positively to the sustainable disposal of solid waste materials. Based on the findings of the study, it was recommended, among others, that enlightenment programs such as awareness creation, town hall meetings, and workshops be organized among community members on the need for sustainable disposal of solid waste materials in Cross River State, Nigeria.

## 1 Introduction

The term “waste” has attracted a wide range of interpretations from different works of life. This is because waste is already a monster with far-reaching negative consequences for both individuals and the environment. Waste, according to Ref. [1], “is any material which has no value for ordinary use” [2]. maintained that “waste is any material or substances including food, leaves, newspapers, bottles, construction debris, candy wrappers, disposables, old cars, or radioactive materials that are not in use by the owner or manufacturer”. However, waste is any solid, liquid, or gaseous substance that has lost its original value to the user at a given time and must be disposed of. The emphasis here is that, to establish a substance or material as a waste means that the substance has lost its economic value or lacks the potential to be productive or valuable to individuals. Waste as a substance consists of solid, liquid, and gaseous. Nevertheless, solid waste is considered in the study [3]. examined “solid wastes as a non-liquid material arising from domestic, commercial, agricultural, and industrial activities”. Nonetheless, solid waste is seen as a non-liquid waste substance lacking the direct value of the user at a particular time and must be sustainably disposed of.

Sustainable disposal of solid waste has been examined by scholars in different disciplines ranging from environmental sciences, sociology, medical sciences, and social sciences, among others. According to Ref. [4], “SDSWM involves the handling of solid waste in an environmentally sustainable way to avoid littering or polluting the ecosystem.” The term “handling” means “to manage something appropriately”. Waste segregation at the source point, transportation, and disposal are all examples of management in this context. However, sustainable disposal of solid waste is seen as the adequate and effective dumping of solid waste materials at the appropriate dump sites (designated waste bins) by individuals without littering the environment.

Sustainable disposal of solid waste material is gradually becoming a difficult task locally and globally with an increase in population, urbanization, and high consumption patterns, among others [5]. maintained that “solid waste materials such as plastics, sachets of water, broken ceramics are discharged indiscriminately into any available space in the environment without considering the negative impacts waste has on the environment.” [6] further maintained that “the negative impacts of indiscriminate disposal of solid waste create unsightly scenes which degenerate into poor environmentally related diseases such as malaria, cholera, typhoid fever, and diarrhea, among others.” Furthermore, despite the health implications for individuals in both urban and rural areas of Cross River State as a result of the increased quantity of indiscriminate disposal of waste materials in the environment, individuals still lack a sustained, organized system for storage, collection, transportation, and hygienic management of solid waste materials [7]. opined that “waste materials are disposed at roadsides, water bodies, open spaces such as market and residential points, anywhere and anyhow” in Cross River State [8]. maintained that “open refuse dumps serve as breeding grounds for disease-bearing organisms such as rats, mosquitoes, cockroaches, and flies”. In spite of these associated problems with solid waste materials, the issue of sustainable disposal of solid waste is lacking in Cross River State. One could ask rhetorical questions such as: are individuals not aware of the prevalence of dangers associated with indiscriminate disposal of solid waste in the environment? Is there no government or private agency responsible for creating awareness about the environment? Irrespective of the associated problems in the environment, there is therefore a need to improve individuals' knowledge of solid waste through awareness creation.

Awareness-creation is of immense importance in encouraging individuals to contribute to sustainable waste disposal [9]. Awareness creation, according to Ref. [10], “is the dissemination of information to individuals through various means, such as television, radio, advertisements in newspapers, billboards, and the use of leaflets.” [10] further maintained that “there is a need for individuals to be aware of the dangers of indiscriminate disposal of solid waste in the environment”. This need could be in the form of public enlightenment campaigns, posters, handbills, among others, in strategic areas or locations in a given community. The need to contribute meaningfully to a community, as some cultures demand in some African countries, is not limited to any form of gender.

The need to integrate gender (male and female) in solid waste disposal is important. It has been viewed by researchers [11,12] that “women have the sole responsibility of managing household waste more than men, partly because they are the managers of the home and consequently the key generators of household waste”. Despite women's responsibilities in managing household waste in Nigeria, there is an argument that women are not given marginal attention in waste management policies and strategies. A research work conducted in Bauchi, Nigeria, for instance, “shows that even though women dominate in waste generation, storage, and collection than men in the area, they are not integrated into the solid waste management system [12]." Nevertheless, the different contributions of males and females in waste management are of great importance in building urban and rural communities.

While urbanization progresses, solid waste resources are becoming a significant public health and environmental issue in both urban and rural areas in many developed countries. The problem is weighty, especially in metropolitan (urban) areas, where variables such as food habits, housing conditions, among others, are increasing [13]. [14] maintained that “plastic, paper, glass, metal, chemicals, and food, among others, constitute a major portion of solid waste in urban areas, thereby polluting the environment to a large quantity, while agricultural waste such as vegetables and leaves is found in rural areas” [15]. posited that “all aspects of man's economic activities involve the generation of waste which is capable of polluting decisions such as monitoring waste generation at source, ensuring adequate the environment.” [16] concluded that “individuals must take proactive disposal of solid waste materials at designated points, and ensuring proper segregation of waste before disposal to avoid environmental pollution."

The theoretical underpinnings of the study are “The System Theory (ST)" proposed by Bertalanffy L. Von in 1969 [17] and " Douglas McGregor's Theory (MT) X and Y″ proposed by McGregor in 1960 [18]. System theory is an integration of different parts of a system in problem solving, and the problem of a system cannot be solved if it is considered in isolation from the different components that make up the system. However, for a system (sustainable disposal of solid waste) to succeed in an environment, it must integrate with other sub-systems (awareness creation) in problem solving. Consequently, theory X of McGregor's theory maintains that the average worker is by nature indolent, dislikes responsibility and prefers to be led, while theory Y has it that individuals are inherently happy to work, they want to exert themselves with objective responsibilities. This implies that, though some individuals are lazy, dislike responsibility and prefer to be led by providing punishable sanctions, some individuals are creative to solve the problem of indiscriminate disposal of solid waste materials. Hence, theory “Y" doesn't involve rewards and punishments for disposing of solid waste. These theories are therefore complementary in upholding this study, such that the researcher anticipates environmental workers' knowledge of the impact of awareness creation on sustainable disposal of solid waste. Resulting to various studies on the way forward [19], conducted a study on the different strategies in ensuring adequate and effective solid waste management [20]. investigated strategies such as replacements of refuse receptacles, staff retraining [21], examined a study on Autoignition behavior and emission of biodiesel from palm oil, waste cooking oil, tyre pyrolysis oil, algae and jatropha among others. Furthermore [22], conducted a study on gender and urban environment on the willingness for waste disposal. These studies to the knowledge of the researcher have not yielded significant results in addressing the issue of solid waste disposal. The absence of this study in the area of the study has provided a gap. Hence, the present study seeks to address environmental education strategies for sustainable disposal of solid waste materials in Cross River State, Nigeria.

### 1.1 Common questions

• What are the mean ratings of urban and rural respondents on the extent to which environmental workers' knowledge of the impact of awareness creation can contribute to the sustainable disposal of solid waste materials in Cross River State?
• What are the mean ratings of male and female respondents on the extent to which environmental workers' knowledge of the impact of awareness creation can contribute to the sustainable disposal of solid waste materials in Cross River State?

### 1.2 Hypotheses

The following null hypotheses were proposed for this research work and tested at a 0.05 level of significance.

• **H0**_**1**_**:** There is no significant difference in the mean ratings of urban and rural respondents on the extent to which environmental workers' knowledge of the impact of awareness creation can contribute to the sustainable disposal of solid waste materials in Cross River State.
• **H0**_**2**_: There is no significant difference in the mean ratings of male and female respondents on the extent to which environmental workers' knowledge of the impact of awareness creation can contribute to the sustainable disposal of solid waste materials in Cross River State.

## 2 Materials and methods

The sequential explanatory mixed methods (qualitative and quantitative) design was adopted in the study. According to Ref. [23], “sequential explanatory” is a type of mixed-method design that includes the compilation and analysis of quantitative data accompanied by the processing and analysis of qualitative data. The sequential explanatory emphasizes quantitative analysis (e.g., the researcher made use of the descriptive survey design in the quantitative aspect), which a researcher follows or supports with the qualitative measure (Focus Group Discussion) to help explain the quantitative findings. This design is consequently considered suitable for this research work because the researcher made use of investigation, collection of both data and assessing information to determine sociodemographic factors and environmental workers' knowledge of the impact of awareness creation on sustainable disposal of solid waste in Cross River State, Nigeria. This study was conducted in Cross River State. Cross River State is one of the 36 states of the Federal Republic of Nigeria. The state is bordered by Benue State in the north, Ebonyi and Enugu States in the west, the Republic of Cameroon in the east, Abia and Akwa Ibom States in the southwest, and the Atlantic Ocean in the south. It is located between latitudes 6°53 and 4°27 north of the equator and between longitudes 7°50 and 9°28 east of the Greenwich Meridian. The state is divided into three (3) senatorial districts, namely the Northern, Central, and Southern Senatorial Districts. The state is politically delineated into eighteen (18) Local Government Areas; these are Akpabuyo, Abi, Akamkpa, Boki, Biase, Bakassi, Calabar Municipality, Calabar South, Bekwarra, Ogoja, Etung, Ikom, Odukpani, Obubra, Obanliku, Yala, Obudu, and Yakurr Local Government Areas. The population of the study comprised 331 respondents, consisting of 120 staff from the Cross-River State Ministry of Environment and 211 government-approved service providers in the Ministry of Environment. The Ministry of Environment comprises 11 departments across the state and government-approved service providers responsible for waste disposal as at the time of this investigation. **(Source: Cross River State Ministry of Environment, Planning, Research and Statistics, 2022;** Appendix A) Ethical approval was issued from the department of Social Science Education, University of Nigeria, Nsukka to all participant of the research **(**Appendix B**)**. The entire population of 331 respondents was used for the study since it was manageable by the researcher. However, the total enumeration sampling technique was used. For the Focus Group Discussion Schedule (FGDS), the researcher purposively sampled 15 respondents in the 5 different locations (Calabar, Ikom, Ogoja, Ugep, and Obudu location) in Cross River State, Nigeria. The sampled respondents comprised 2 staff of the Ministry of Environment and 1 government-approved service provider. The choice of the population for the study is based on the fact that all the staff of the Ministry of Environment and the government-approved service providers are responsible for creating awareness about waste disposal and educating the populace on sustainable ways of disposing of solid waste materials, among others. The instruments that were used for data collection were a structured questionnaire titled, “Questionnaire on Sociodemographic Factors and Environmental Workers’ Knowledge of the Impact of Awareness Creation on Sustainable Disposal of Solid Wastes (QSFEWKIACSDSW) and the Focus Group Discussion Schedule (FGDS) **(**Appendix C**)**.

The structured questionnaire was divided into two parts. The first part requested respondents to provide sociodemographic information such as location and gender, while the second part comprised 9 items on a 5-point Likert-Type scale measuring sociodemographic factors and environmental workers’ knowledge of the impact of awareness creation on sustainable disposal of solid waste. The questionnaire was structured on a 5-point rating scale of Very high extent (VHE) 4.50–5.00, High extent (HE) 3.50–4.49, Moderate extent (ME) 2.50–3.49, Low extent (LE) 1.50–2.49, and very low extent (VLE) 1.00–1.49. The Focus Group Discussion Schedule (FGDS) was employed to support the findings of the quantitative aspect. The FGDS was based on awareness-creation items developed by the researcher. The instruments (qualitative and quantitative) were face validated by experts, two from the Department of Social Science Education (Geography and Environmental Education unit) and one from the Measurement and Evaluation Unit of the Department of Science Education, all in the Faculty of Education, University of Nigeria, Nsukka.

### 2.1 Data analysis

The mean and standard deviation were used to answer the research questions while the independent sample *t*-test was computed for data analysis. Reliability was determined by computing the internal consistency of the responses to question items on the instrument using the coefficient value of alpha [24]. Furthermore, a statistically significant difference was computed between variables by using the independent *t*-test analysis.

## 3 Findings

### 3.1 Reliability and validity of scale scores

The face validity of the instrument used was ascertained by three senior academics in the University of Nigeria, Nsukka within the fields of geography and environmental education and measurement and evaluation to ascertain the suitability and relevance of each proposed item on the questionnaire. The reliability coefficient is presented below (Table 1).

**Table 1:** Alpha value for the items in the instrument (QSFEWKIACSDSW) used for the study.

Case Processing Summary			
		N	%
Cases	Valid	30	100.0
	Excludeda	0	.0
	Total	30	100.0

Reliability Statistics		
Cronbach's Alpha	Cronbach's Alpha Based on Standardized Items	N of Items
.577	.581	9

Item Statistics			
	Mean	Std. Deviation	N
Awareness Creation	4.60	.621	30
Awareness Creation	4.37	.556	30
Awareness Creation	4.50	.572	30
Awareness Creation	4.07	1.015	30
Awareness Creation	3.67	1.155	30
Awareness Creation	4.30	.877	30
Awareness Creation	4.43	.679	30
Awareness Creation	3.77	.858	30
Awareness Creation	3.90	.923	30

	Mean	Minimum	Maximum	Range	Maximum/Minimum	Variance	No of Items
Item Means	4.178	3.667	4.600	.933	1.255	.115	9
Item Variances	.689	.309	1.333	1.024	4.312	.123	9
Inter-Item Correlations	.133	−.336	.549	.886	−1.633	.049	9

## 4 Results

Results in Table 2 present the mean ratings of urban and rural respondents on the extent to which environmental workers’ knowledge of the impact of awareness creation can contribute to the sustainable disposal of solid waste materials. Indicates that respondents from urban areas had mean ratings within the range of 4.50–5.00 set as a criterion for very high extent on items 1, 2 and 6, while items 3, 4, 5, 7, 8 and 9 had mean ratings within the range of 3.50–4.49 set as a criterion for high extent. Similarly, respondents from rural areas had mean ratings within the range of 4.50–5.00 set as a criterion for very high extent on items 1, 2, 6, and 9, whereas items 3, 4, 5, 7, and 8 had mean ratings within the range of 3.50–4.49 set as a criterion for high extent. Furthermore, respondents from urban areas had a grand mean rating of 4.34 with a standard deviation of 0.41, while those from rural areas had a grand mean rating of 4.39 with a standard deviation of 0.42, which is still within the range of 3.50–4.49 set as a criterion for high extent. This is an indication that both urban and rural respondents were of the view that awareness creation can contribute to the sustainable disposal of solid waste materials in urban and rural areas of Cross River State to a high extent.Where, very high extent (VHE) equals 4.50–5.00, High extent (HE) equals 3.50–4.49, Moderate extent (ME) equals 2.50–3.49, Low extent (LE) equals 1.50–2.49 while very low extent (VLE) equals 1.00–1.49.

**Table 2:** Location sociodemographic factor in environmental workers’ knowledge of the impact of awareness creation on sustainable disposal of solid waste materials in Cross River State. N = 331.

S/N	Items	Urban		Decision	Rural		Decision
		Mean	SD		Mean	SD	
1.	Adequate enlightenment prepares people to understand the health implications of indiscriminate disposal of solid waste materials	4.75	.57	VHE	4.82	.44	VHE
2.	Promoting environmental consciousness helps in the control of indiscriminate disposal of solid waste materials	4.67	.75	VHE	4.79	.54	VHE
3.	Raising people's environmental consciousness helps in the control of indiscriminate dumping of solid waste in gutters and drainage systems	4.39	.77	HE	4.45	1.03	VHE
4.	Environmental enlightenment increases people's understanding that solid waste dumped in drainage channels causes flooding	4.15	1.25	HE	3.93	1.39	HE
5.	Increasing environmental consciousness through environmental awareness creation reduces incineration of solid waste as a means of disposal	4.00	1.00	HE	3.95	1.26	HE
6.	Adequate environmental sensitization promotes people's disposal of solid waste at designated waste dump sites	4.57	.81	VHE	4.58	.76	VHE
7.	Adequate sensitization can discourage indiscriminate disposal of solid waste in streams and rivers	4.34	1.08	HE	4.20	1.22	HE
8.	Proper sensitization promotes the act of segregating waste before disposal among people	3.87	1.08	HE	4.19	1.09	HE
9.	Fostering appropriate knowledge about the environment promotes sustainable disposal of solid waste material	4.34	.90	HE	4.65	.78	VHE
	Grand Mean	4.34	.41	HE	4.39	.42	HE

### 4.1 Differences in Cross River State Ministry of Environment and government-approved service providers' responses by location

Table 3 shows the mean difference between urban and rural dwellers on the extent to which awareness creation can contribute to the sustainable disposal of solid waste materials in Cross River State. The result shows that a t-value of −0.995 at 329° of freedom with an exact probability (sig.) value of 0.321 was obtained. The null hypothesis was therefore upheld since the exact probability (sig.) value of 0.321 is greater than the 0.05 level of significance. Thus, there is no significant difference in the mean ratings of urban and rural respondents on environmental workers’ knowledge of the impact of awareness creation on sustainable disposal of solid waste materials. This implies that both urban and rural respondents' concord that awareness creation can contribute to the sustainable disposal of solid waste materials in Cross River State to a very high extent.

**Table 3:** Results of urban and rural respondents on environmental workers’ knowledge impact of awareness creation on sustainable disposal of solid waste materials in Cross River State.

Location	No. of Respondents	$\bar{X}$	S. D	Df	t-val.	Sig. (2-tailed)	Decision
Urban	246	4.34	.41	329	−.995	.321	Not Rejected
Rural	85	4.39	.42				

This result is buttressed by the findings from the FGDs held on the 24th, 26th, 30th of September 3rd, and October 8, 2019 with the staff of the Ministry of Environment and government-approved service providers. From the FGD, discussants were of the view that awareness creation can improve sustainable disposal of solid waste materials through educating the populace on the dangers of indiscriminate disposal of solid waste materials. Adequate sensitization of individuals should be carried out in schools, churches, mosques, courts, among market women, drivers, fishermen, farmers, town halls, village squares, clubs, among others on the dangers of indiscriminate dumping of solid waste materials. Remediation measures should be provided to both urban and rural communities through the organizing of seminars, talk shows, with short dramas in schools, marketplaces, villages, among others on the hazards associated with indiscriminate disposal of solid waste materials. From the respondents, the discussions were to a high extent that awareness creation can contribute to the sustainable disposal of solid waste materials in urban and rural areas of Cross River State.

### 4.2 Descriptive statistics

Results in Table 4 present the mean ratings of males within the range of 4.50–5.00 set as a criterion for very high extent on items 1, 2 and 6, while items 3, 4, 5, 7, 8 and 9 had mean ratings within the range of 3.50–4.49 set as a criterion for high extent. Similarly, female respondents had mean ratings within the range of 4.50–5.00 set as a criterion for very high extent on items 1, 2, 6, and 7, whereas items 3, 4, 5, 8, and 9 had mean ratings within the range of 3.50–4.49 set as a criterion for high extent. Furthermore, male respondents had a grand mean rating of 4.36 with a standard deviation of 0.43 while female respondents had a grand mean rating of 4.35 with a standard deviation of 0.37, which is still within the range of 3.50–4.49 set as a criterion for high extent. This is an indication that both male and female respondents were of the view that awareness creation can contribute to the sustainable disposal of solid waste materials among male and female respondents in Cross River State to a high extent.

**Table 4:** Gender and sociodemographic factors in environmental workers’ knowledge of the impact of awareness creation on sustainable disposal of solid waste materials in Cross River State N = 331.

S/N	Items	Male		Decision	Female		Decision
		Mean	SD		Mean	SD	
1	Adequate enlightenment prepares people to understand the health implications of indiscriminate disposal of solid waste materials	4.75	.56	VHE	4.81	.48	VHE
2	Promoting environmental consciousness helps in the control of indiscriminate disposal of solid waste materials	4.71	.71	VHE	4.67	.67	VHE
3	Raising people's environmental consciousness helps in the control of indiscriminate dumping of solid waste in gutters and drainage systems	4.48	.85	HE	4.17	.79	HE
4	Environmental enlightenment increases people's understanding that solid waste dumped in drainage channels causes flooding	4.06	1.30	HE	4.21	1.23	HE
5	Increasing environmental consciousness through environmental awareness creation reduces incineration of solid waste as a means of disposal	4.00	1.09	HE	3.96	1.02	HE
6	Adequate environmental sensitization promotes people's disposal of solid waste at designated waste dump sites	4.56	.791	V HE	4.62	.815	VHE
7	Adequate sensitization can discourage indiscriminate disposal of solid waste in streams and rivers	4.22	1.18	HE	4.57	.85	VHE
8	Proper sensitization promotes the act of segregating waste before disposal among people	4.01	1.12	HE	3.75	.96	HE
9	Fostering appropriate knowledge about the environment promotes sustainable disposal of solid waste material	4.43	.96	HE	4.38	.60	HE
	Grand Mean	4.36	.43	HE	4.35	.37	HE

Where, Very high extent (VHE) equals 4.50–5.00, High extent (HE) equals 3.50–4.49, Moderate extent (ME) equals 2.50–3.49, Low extent (LE) equals 1.50–2.49 while Very low extent (VLE) equals 1.00–1.49.

### 4.3 Gender differences in responses from the Cross River State Ministry of Environment and government-approved service providers

Table 5 shows the mean difference between male and female respondents on environmental workers’ knowledge of the impact of awareness creation on sustainable disposal of solid waste materials in Cross River State. The result shows that a t-value of. 159 at 329° of freedom with an exact probability (sig.) value of. 874 was obtained. The null hypothesis was therefore upheld since the exact probability (sig.) value of. 874 is greater than the 0.05 level of significance. Thus, there is no significant difference in the mean ratings of male and female respondents on the extent to which awareness creation can contribute to the sustainable disposal of solid waste materials in Cross River State. This implies that both male and female respondents' concord that awareness creation can contribute to sustainable disposal of solid waste materials in Cross River State to a very high extent.

**Table 5:** Results of male and female respondents on environmental workers’ knowledge impact of awareness creation on sustainable disposal of solid waste materials in Cross River State.

Gender	No. of Respondents	$\bar{X}$	S. D	df	t-val.	Sig. (2-tailed)	Decision
Male	250	4.36	.43	329	.159	.874	Not Rejected
Female	81	4.35	.37				

This result is buttressed by the findings from the Focused Group Discussion held on the 24th, 26th, and 30th of September, the 3rd, and October 8, 2019 with the staff of the Ministry of Environment and government-approved service providers. From the FGD, discussants were of the view that awareness creation can improve sustainable disposal of solid waste materials through mass media (television, radio, jingles) and also educate the public on how to sort waste at a source point and differentiate between biodegradable and non-biodegradable waste. The inclusion of environmental waste management subjects should be domiciled in the school curriculum from primary, secondary, and tertiary education. This will help create effective awareness among students and integrate the concept of waste disposal into their behavior. Enlightenment campaigns through public address systems (radio, television, and town cries, among others) should be used to educate the public on the dangers of keeping waste within their environment rather than disposing of the waste in the appropriate quarters because waste itself is hazardous to human existence. From the respondents, the discussions were to a high extent that awareness creation can contribute to the sustainable disposal of solid waste materials among males and females in Cross River State.

## 5 Discussion

This paper presents the discussion in this chapter according to the research questions and hypotheses. Based on these discussions, conclusions are drawn, and recommendations made.

The result of findings in research question one showed that to a high extent, awareness creation can contribute to sustainable disposal of solid waste materials among male and female in Cross River State. Information from both qualitative and quantitative data indicated that awareness creation can improve sustainable disposal of solid waste materials through; mass media (television, radio, jingles) and also educating the public on how to sort waste at source point and differentiate between biodegradable and non-biodegradable waste, the inclusion of environmental waste management subjects should be domicile in school curriculum from primary, secondary and tertiary education. This will help create effective awareness among students and integrate the concept of waste disposal in their behavior and enlightenment campaign through public address systems (radio, television, town cries among others) should be used by educating the public on the dangers of keeping waste within their environment rather than disposing the waste in the appropriate quarters because waste itself is hazardous to human existence. From the respondents, the discussions were to a high extent that awareness creation can contribute to sustainable disposal of solid waste materials among male and female in Cross River State. The result further reveled that there was no significant difference in the mean ratings of male and female respondents on the extent to which awareness creation can contribute to sustainable disposal of solid waste materials in Cross River State. The finding is in line with [10] which indicated that awareness creation can improve sustainable disposal of solid waste materials through dissemination of informing to individuals (male and female) such as television, radio, advertisements in newspapers, billboards, use of leaflet, enlightenment campaigns, clean up campaigns among others. In addition [25], stipulated that awareness creation strategy would clearly indicate commitment to raising public awareness on sustainable disposal of solid waste material in the environment among male and female in Cross River State.

However, the result of findings in research question one showed that to a high extent, awareness creation can contribute to sustainable disposal of solid waste materials in urban and rural areas of Cross River State. Information from both qualitative and quantitative data indicated that awareness creation can improve sustainable disposal of solid waste materials through, educating the populace on the dangers of indiscriminate disposal of solid waste materials. Adequate sensitization of individuals should be carried out in schools, churches, mosques, court, among market women, drivers, fisher men, farmers, town halls, village squares, clubs among others on the dangers of indiscriminate dumping of solid waste materials and remediation measures should be provided to both urban and rural community and organizing of seminars, talk shows with short dramas in schools, market places, villages among others on the hazards associated with indiscriminate disposal of solid waste materials. From the respondents, the discussions were to a high extent that awareness creation can contribute to sustainable disposal of solid waste materials in urban and rural areas of Cross River State. The result further reveled that there was no significant difference in the mean ratings of urban and rural respondents on the extent to which awareness creation can contribute to sustainable disposal of solid waste materials in Cross River State. The finding is in line with [26] which posited that; awareness should be created among residents to manage household refuse and educate individuals on the hazards that indiscriminate disposal of waste materials could pose to the environment and to inhabitants. Furthermore [25], maintained that awareness creation could also be carried out through advertisements on newspapers, television, radio, billboards, use of leaflet, campaigns among others on the importance of sustainable disposal of solid waste material to the environment as well as human health [10]. Omran et al. further maintained that the integration of all the awareness strategies (advertisements on newspapers, television, radio, billboards, use of leaflet, campaigns) can increase public participation in sustainable disposal of solid wastes materials.

## 6 Conclusion

From the findings of this study, it is imperative that environmental workers’ knowledge impact of awareness creation on sustainable disposal of solid waste materials in Cross River State be improved in both urban and rural areas-male and female alike. Although there was no significant difference in the scores of urban and rural responses and those of male and female, the qualitative responses indicate the need for adequate enlightenment programs such as awareness creation, seminars, talk shows, among others, that can be achieved to foster sustainable disposal of solid waste material. Also, since every individual is important in protecting the environment through the choices they make, there is a need for everyone to synergize with authorities in ensuring sustainable disposal of solid waste materials.

Furthermore, studies should incorporate other environmental education strategies such as community participation in addressing solid waste materials in addition to awareness creation. A wider study area, along with a larger sample size, is also suggested to present a broader picture of the status quo in environmental workers’ knowledge impact of awareness creation on sustainable disposal of solid waste materials.

## Author contribution statement

UBA JAMES UBA: Conceived and designed the experiments; Performed the experiments; Analyzed and interpreted the data; Contributed reagents, materials, analysis tools or data; Wrote the paper.

## Data availability statement

The data that has been used is confidential.

## Additional information

Supplementary content related to this article has been published online at [URL].

## Declaration of competing interest

The authors declare that they have no known competing financial interests or personal relationships that could have appeared to influence the work reported in this paper
