# Supplementary material for: Sociodemographic factors and environmental workers’ knowledge of the impact of awareness creation on sustainable disposal of solid wastes
Source: Heliyon. 2023 Jul 17;9(7):e18122. doi: 10.1016/j.heliyon.2023.e18122 (PMC10393608; doi:10.1016/j.heliyon.2023.e18122)
Supplement: Multimedia component 1 [file mmc1.docx]

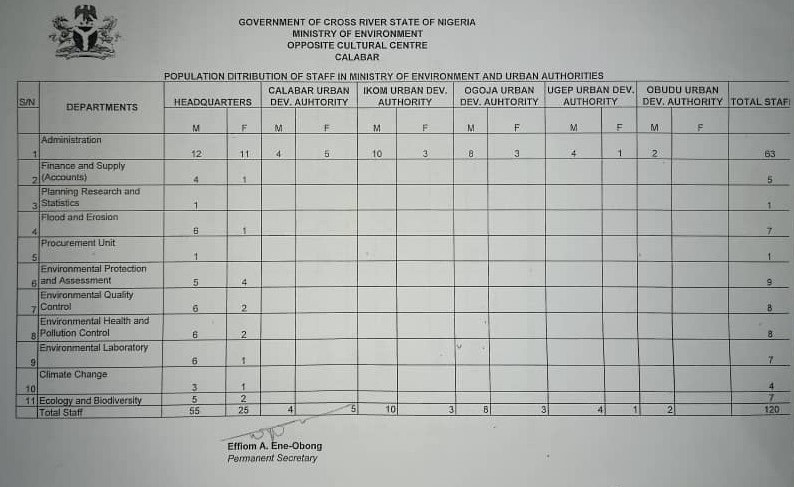

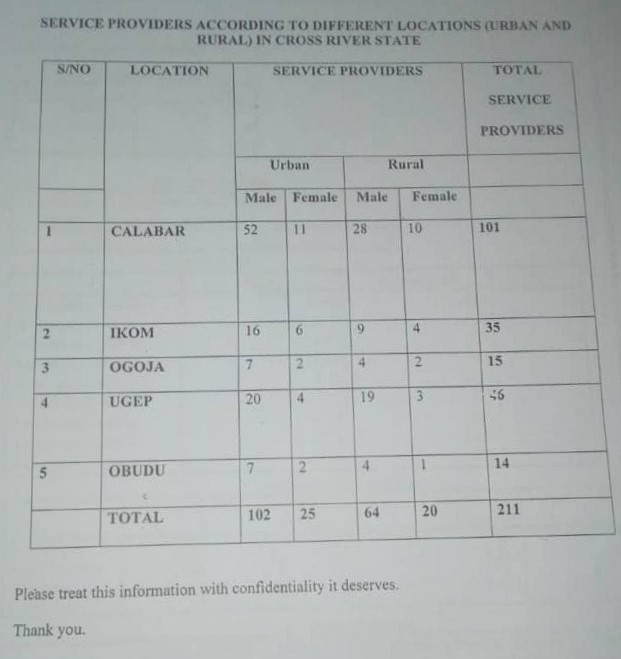


**Appendix A: Cross River State Ministry of Environment, Planning Research and Statistics, 2022.**

**Appendix B: Ethical Approval**


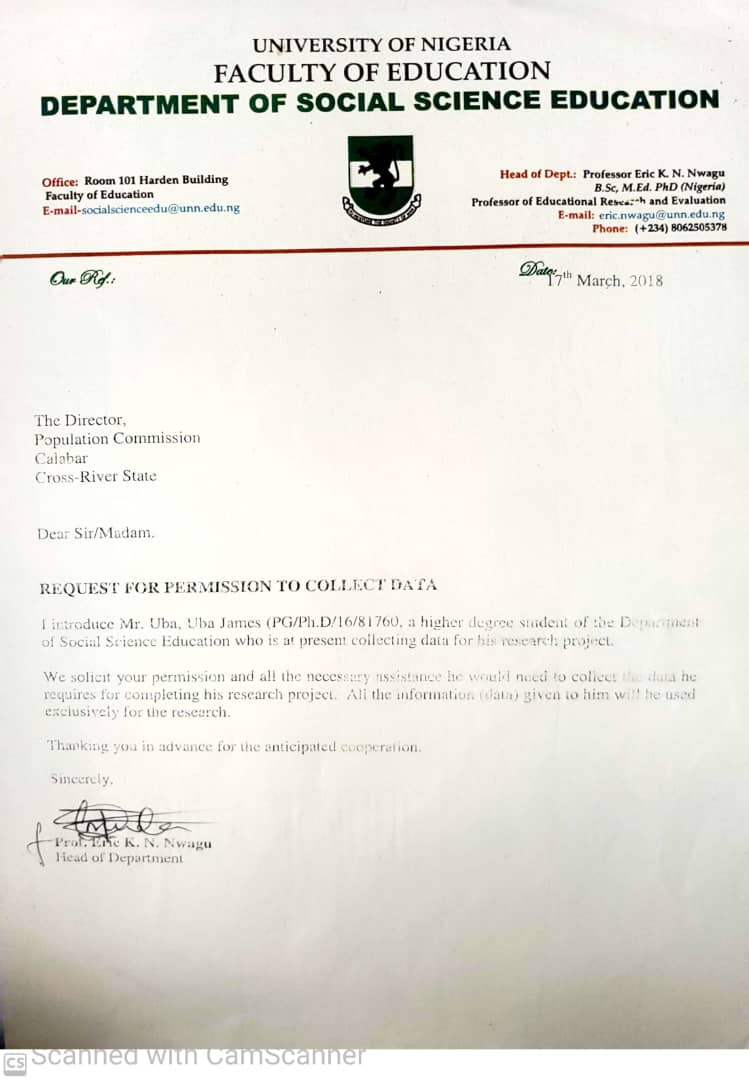


**APPENDIX C: Questionnaire on Sociodemographic Factors and Environmental Workers’ Knowledge of the Impact of Awareness Creation on Sustainable Disposal of Solid Wastes (QSFEWKIACSDSW)**

**Instruction:** Please respond to the under listed questions by a tick [√] where appropriate.

**Section A: Demographic Characteristics of Respondent**

1. Location: (a) Urban [] (b) Rural [ ]
2. Gender: (a) Male [ ] (b) Female [ ]

**SECTION B**

**Instruction**: Tick [√] the option that most represent your opinion on each item of the questionnaire

Response Weight

VHE: Very high extent (5- points)

HE: High extent (4-points)

ME: Moderate Extent (3-Points)

LE: Low extent (2-points)

VLE: Very low extent (1-point)

| **S/No** | **Item Statement** | **VHE** | **HE** | **ME** | **LE** | **VLE** |
| --- | --- | --- | --- | --- | --- | --- |
| 1 | Adequate enlightenment prepares people to understand the health implications of indiscriminate disposal of solid waste materials |  |  |  |  |  |
| 2 | Promoting environmental consciousness helps in the control of indiscriminate disposal of solid waste materials |  |  |  |  |  |
| 3 | Raising people’s environmental consciousness helps in the control of indiscriminate dumping of solid waste in gutters and drainage systems |  |  |  |  |  |
| 4 | Environmental enlightenment increases people’s understanding that solid waste dumped in drainage channels causes flooding |  |  |  |  |  |
| 5 | Increasing environmental consciousness through environmental awareness creation reduces incineration of solid waste as a means of disposal |  |  |  |  |  |
| 6 | Adequate environmental sensitization promotes people’s disposal of solid waste at designated waste dump sites |  |  |  |  |  |
| 7 | Adequate sensitization can discourage indiscriminate disposal of solid waste in streams and rivers |  |  |  |  |  |
| 8 | Proper sensitization promotes the act of segregating waste before disposal among people |  |  |  |  |  |
| 9 | Fostering appropriate knowledge about the environment promotes sustainable disposal of solid waste material |  |  |  |  |  |

**Focus Group Discussion Questions**

1. What do you understand by sustainable disposal of solid waste materials?
2. In what ways can awareness creation improve sustainable disposal of solid waste materials?
3. In what ways can community participation improve the sustainable disposal of solid waste materials?
4. In what ways can the establishment of environmental clubs improve the sustainable disposal of solid waste materials?
5. In what ways can legal institutions improve the sustainable disposal of solid waste materials?
6. In what ways can NGOs improve the sustainable disposal of solid waste materials?
